# A Rare Case of Unilateral Morning Glory Disc Anomaly in a Patient with Turner Syndrome: Report and Review of Posterior Segment Associations

**DOI:** 10.1155/2018/5969157

**Published:** 2018-06-28

**Authors:** Dev R. Sahni, Michael Wallace, Mansi Kanhere, Hind Al Saif, Natario Couser

**Affiliations:** ^1^Virginia Commonwealth University School of Medicine, Richmond, VA, USA; ^2^Department of Ophthalmology, Virginia Commonwealth University School of Medicine, Richmond, VA, USA; ^3^Department of Pediatrics, Division of Endocrinology, Virginia Commonwealth University School of Medicine, Richmond, VA, USA; ^4^Department of Pediatrics, Division of Clinical Genetics, Virginia Commonwealth University School of Medicine, Richmond, VA, USA

## Abstract

Turner syndrome is a common sex chromosome disorder affecting females. The disorder is caused by a partial loss, complete absence, or structural abnormality of one X chromosome. The clinical presentation is broad and ranges from the classic phenotype to minimal clinical manifestations. Ocular abnormalities associated with the syndrome are common. Reports describing abnormal eye features in individuals with Turner syndrome generally involve refractive errors (myopia or hyperopia), strabismus, and external or anterior segment abnormalities including hypertelorism, epicanthal folds, and ptosis. Posterior ocular segment anomalies involving the optic nerve and retina in Turner syndrome have been rarely reported. We report a rare presentation of an 11-year-old female with Turner syndrome and unilateral morning glory disc anomaly.

## 1. Introduction

Turner syndrome, first described by the physician Henri Turner in 1938, is a sex chromosome disorder caused by a partial loss, complete absence, or structural abnormality of one X chromosome in females. It has been estimated to occur in 1:2000 of live female births [1]. Ocular abnormalities involving the anterior ocular segment, eyelids, external ocular adnexa, and refractive status of the eye in patients with Turner syndrome are not infrequent [1]. Dedicated reports of the association between Turner syndrome and ocular abnormalities date back to the 1960s [2, 3]. Abnormalities commonly involving the eye include refractive errors (myopia or hyperopia), amblyopia, strabismus (esotropia or exotropia), and external and anterior segment abnormalities including hypertelorism, epicanthus, downslanting palpebral fissures, and ptosis [1]. Less common ocular abnormalities include blue sclera, congenital glaucoma, convergence insufficiency nystagmus, decreased accommodation, hypertelorism, presenile cataracts, and red-green color deficiency [1, 4]. Reports of co-occurring Turner syndrome and abnormalities of the optic nerve and retina are rare.

We describe an 11-year-old female who presented with strabismus and amblyopia in her right eye secondary to a morning glory disc anomaly [5]. The morning glory disc anomaly (MGDA) was first described by Kindler in 1970 [5]. The term MGDA describes a congenital optic disc malformation consisting of radial retinal vessels, peripapillary pigmentation, and a central glial tuft [5]. This pathological insult often results in severely decreased visual acuity [5].

## 2. Case Report

**Figure 1:**
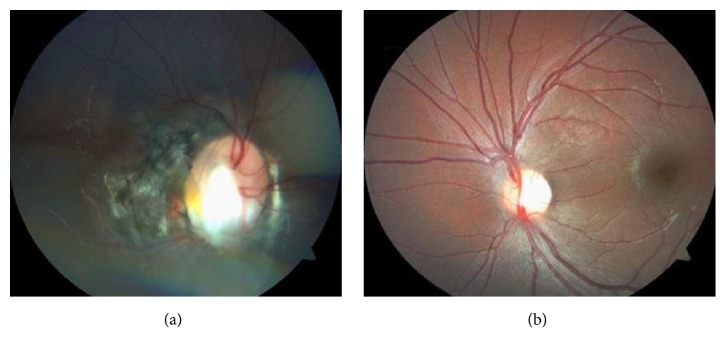
Large anomalous optic disc with conical excavation, significant peripapillary pigmentation, and some straightening of the retinal vessels arising from the disc margin of the right eye (a); normal appearing fundus of the left eye (b).

An 11-year-old female with Turner syndrome (45, X) presented to the eye clinic with strabismus and poor vision in the right eye. The patient was of short stature and had a webbed neck. Ophthalmic examination was remarkable for a visual acuity of counting fingers in the right eye and 20/20 in the left eye, 1+ right afferent pupillary defect, and having a constant esotropia of 15 prism diopters. Stereopsis was absent. Hypertelorism was present. The anterior segment was unremarkable. The optic nerve in the right eye was large in appearance with central excavation and extensive peripapillary pigmentation; some straightening of the retinal vessels arising from the disc margin was present (Figure 1(a)). The left optic disc appeared normal in size and was pink with a normal appearing cup and sharp disc margins (Figure 1(b)).

## 3. Discussion

To our knowledge, this case represents the first report of Turner syndrome associated with MGDA. Co-occurring Turner syndrome and posterior segment abnormalities involving the retina and optic nerve have been rarely reported. Further investigation can help determine if the chromosomal abnormality may be responsible for these rare co-occurrences.

Yanagisawa and Yokoyama in 1975 described a female diagnosed with isochromosome X mosaicism; myopia and optic nerve atrophy were associated [6]. Mason and Tasman in 1996 described a 2-month-old infant referred to an ophthalmologist for bilateral leukocoria and absence of response to visual stimuli [7]. The exam was notable for the presence of shallow anterior chambers, posterior synechiae, and retrolental membranes [7]. Ocular echography revealed bilateral V-shaped retinal detachments [7]. Gotoh et al. in 1998 described two separate unrelated cases of female infants diagnosed prenatally with Turner syndrome via amniotic fluid analysis who were noted to have significant retinal abnormalities in early infancy [8]. The first patient was a 4-month-old female born at 34 weeks of gestation and presented with avascular areas, neovascularization, and anastomoses of retinal vessels in the temporal zone in the left eye [8]. The second patient was a 19-week-old female born at 33 weeks of gestation and presented with avascular areas, neovascularization, anastomoses of retinal vessels, and a vitreous hemorrhage within the temporal zone in the right eye [8]. Tsunekawa et al. in 2007 described a 35-year-old patient with Turner syndrome presenting with four months of progressive right metamorphopsia who was ultimately diagnosed with punctate inner choroidopathy [9]. Girguis-Bucher and Schlegel-Wagner in 2013 described a patient with Turner syndrome and a sella meningioma [10]. Prior to transsphenoidal surgery, computed tomography of the brain was performed which revealed an incidental abnormal course of the optic nerve through the floor of the sphenoidal sinus [10]. Chiu et al. in 2018 described a unique case of angioid steaks in a patient with Turner syndrome [11]. This patient presented with decreased and distorted vision in the left eye [11].

We present the first reported case of the morning glory disc anomaly associated with Turner syndrome. Due to optic nerve and retinal involvement with MGDA, this is an additional case of abnormalities associated with the posterior segment in Turner syndrome. The prevalence of MGDA is estimated at 2.6:100000 individuals with a slightly greater female predilection [5]. Strabismus is a common presenting factor for MGDA and should be actively investigated [5, 12]. Further studies will be needed to elucidate a definitive association between Turner syndrome and MGDA. MGDA has been reported in co-occurring cases with Aicardi syndrome, an X-linked condition; therefore chromosomal or other genetic aberrations could be a contributory factor [5, 13–16]. The pathogenesis of MGDA remains elusive, though morphogenesis of the posterior sclera and lamina cribosa has been described [5]. This unique case provides incentive to further investigate the chromosomal disease associations with this rare disc anomaly and highlights the importance of considering the possibility of posterior ocular segment abnormalities in patients with Turner syndrome.
